# CRISPR/Cas9-Mediated Knockout of *GmFATB1* Significantly Reduced the Amount of Saturated Fatty Acids in Soybean Seeds

**DOI:** 10.3390/ijms22083877

**Published:** 2021-04-09

**Authors:** Jing Ma, Shuo Sun, James Whelan, Huixia Shou

**Affiliations:** 1 State Key Laboratory of Plant Physiology and Biochemistry, College of Life Sciences, Zhejiang University, Hangzhou 310058, China; majing1002@zju.edu.cn (J.M.); sunshuo2019@zju.edu.cn (S.S.); j.whelan@latrobe.edu.au (J.W.); 2 The Provincial International Science and Technology Cooperation Base on Engineering Biology, International Campus of Zhejiang University, Haining 314400, China; 3 Australian Research Council Centre of Excellence in Plant Energy Biology, Department of Animal, Plant and Soil Science, School of Life Science, La Trobe University, Bundoora, VIC 3086, Australia; 4 Hainan Institute, Zhejiang University, Sanya 572025, China

**Keywords:** oil composition, acyl-acyl carrier protein thioesterases, CRISPR/Cas9, soybean

## Abstract

Soybean (*Glycine max*) oil is one of the most widely used vegetable oils across the world. Breeding of soybean to reduce the saturated fatty acid (FA) content, which is linked to cardiovascular disease, would be of great significance for nutritional improvement. Acyl-acyl carrier protein thioesterases (FATs) can release free FAs and acyl-ACP, which ultimately affects the FA profile. In this study, we identified a pair of soybean FATB coding genes, *GmFATB1a* and *GmFATB1b*. Mutants that knock out either or both of the *GmFATB1* genes were obtained via CRISPR/Cas9. Single mutants, *fatb1a* and *fatb1b*, showed a decrease in leaf palmitic and stearic acid contents, ranging from 11% to 21%. The double mutant, *fatb1a:1b*, had a 42% and 35% decrease in palmitic and stearic acid content, displayed growth defects, and were male sterility. Analysis of the seed oil profile revealed that *fatb1a* and *fatb1b* had significant lower palmitic and stearic acid contents, 39–53% and 17–37%, respectively, while that of the unsaturated FAs were the same. The relative content of the beneficial FA, linoleic acid, was increased by 1.3–3.6%. The oil profile changes in these mutants were confirmed for four generations. Overall, our data illustrate that *GmFATB1* knockout mutants have great potential in improving the soybean oil quality for human health.

## 1. Introduction

Soybean (*Glycine max*) oil is the most important vegetable oil in the world, accounting for 28% of the world’s edible oil consumption in 2019 [1]. Soybean oil contains essential fatty acids (FAs) for human nutrition. The oil content in soybean seeds is approximately 20% (w/w). It contains five FAs: palmitic acid (C16:0), stearic acid (C18:0), oleic acid (C18:1), linoleic acid (C18:2), and linolenic acid (C18:3), with average contents of 10%, 4%, 18%, 55%, and 13%, respectively [2]. Excessive intake of saturated FAs can lead to elevated blood cholesterol, triglycerides, and low-density lipoprotein cholesterol (LDL-C), which in turn leads to arterial stenosis and atherosclerosis and increase the risk of coronary heart disease [3,4,5]. Oleic acid can reduce the content of total cholesterol and harmful cholesterol in the human body, and thus is considered as a beneficial FA. Linoleic acid is reported to have anti-cancer properties, lower blood lipids, is anti-atherosclerosis, improve immunity, and prevent and treat diabetes [6,7,8]. Linolenic acid is reported to enhance brain vitality, promote healthy aging, and lower blood pressure [9]. However, polyunsaturated FAs, especially linolenic acid, are easily oxidized by lipoxygenase, leading to deterioration and oxidation of soybean oil. Therefore, increasing the content of oleic and reducing the content of saturated FAs and linolenic acid in seeds are important goals of soybean breeding.

The synthesis and accumulation of FAs is a complex physiological and biochemical process, involving the coordinated expression of numerous genes, encoding enzymatic and regulatory functions. In addition, the steps in FA synthesis or recycling takes place in a variety of locations in the cell, such as plastids, endoplasmic reticulum (ER), and cytoplasm. In plants, FAs are exclusively synthesized de novo in plastids. The plastidial pathway of fatty acid biosynthesis consists of two key enzymes, acetyl-CoA carboxylase and fatty acid synthase. Acetyl-CoA carboxylase catalyzes the formation of malonyl-CoA from acetyl-CoA, and fatty acid synthase transfers the malonyl moiety to acyl carrier protein (ACP) and catalyzes the extension of the growing acyl chain with malonyl-ACP. The carbon chain extension can be terminated to release free FAs and ACP by fatty acyl carrier protein thioesterase (FAT) or to produce glycerolipids by the action of the plastid acyltransferase in plastids. The synthesized FAs are transported to the endoplasmic reticulum where glycerolipid synthesis takes place [10,11]. Therefore, FATs play important roles in plastid-localized fatty acid synthase and determine the chain length of de novo synthesized fatty acids.

Plant FATs were classified into FATA and FATB subgroups based on amino acid sequence and substrate specificity [12]. *FATA* encodes 18:l-ACP thioesterase, while *FATB* encodes thioesterases preferring acyl-ACPs that have saturated acyl groups [12,13]. The N-terminus of both FATA and FATB acyl-ACP thioesterases determines the substrate specificity [14]. Functional analysis of these FATs have focused on these enzymes isolated from seed oil-rich species, such as *Persea americana* (avocado mesocarp) [15,16], *Carthamus tinctorius* (safflower) [17,18], *Umbellularia californica* (california bay) [19,20,21,22,23], *Brassica napus* (rapeseed) [24], *Helianthus annuus* (sunflower) [25,26], and *Cuphea hookeriana* (cuphea) [27]. In 1995, the first thioesterase sequence was identified in *Arabidopsis thaliana*, and its thioesterase activity on long-chain acyl ACP was demonstrated [28]. Disruption of the AtFATB led to a significant reduction in the saturated fatty acid contents in leaves and growth retardation [29,30]. Overexpression of *AtFATB* using a seed-specific promoter resulted in accumulation of increased palmitate in seeds [31]. In maize, the palmitic content of mutants with an 11 bp insertion or single nucleotide insertion in *FATB* were reduced [6,32]. Overexpression of *CnFATB3* from coconut in *Arabidopsis* seeds resulted in the increase in saturated FA content [33]. Soybean mutant N0304-303-3, which contains a 254-kb genomic deletion containing 19 genes, including the *FATB1a* gene, have low levels of palmitic acid [34]. However, which gene(s) among the 19 genes plays a key role is not clear.

In this study, we generated CRISPR/Cas9 mutants for the pair of FATB protein coding genes, *GmFATB1a* and *GmFATB1b*. The single gene mutants, *fatb1a* or *fatb1b*, showed a significant decrease in palmitic acid and stearic acid content in both leaves and seeds. The double mutant *fatb1a:1b* resulted in a greater reduction in palmitic acid and stearic acid content than that in the single mutants, displayed growth defects, and had male sterility. Analysis of the seed oil revealed that *fatb1a* and *fatb1b* significantly reduced the content of the two saturated FAs, palmitic acid and stearic acid, compared with the contents of the wild type (WT) plants in soybean seeds. In terms of the relative composition of soybean oil, *fatb1a* and *fatb1b* mutations increased the content of the beneficial FAs and reduced the unhealthy FAs. This provides the molecular identity of the targets to improve the nutritional quality of soybean oil.

## 2. Results

### 2.1. Expression of GmFATB1a and GmFATB1b

**Figure 1:**
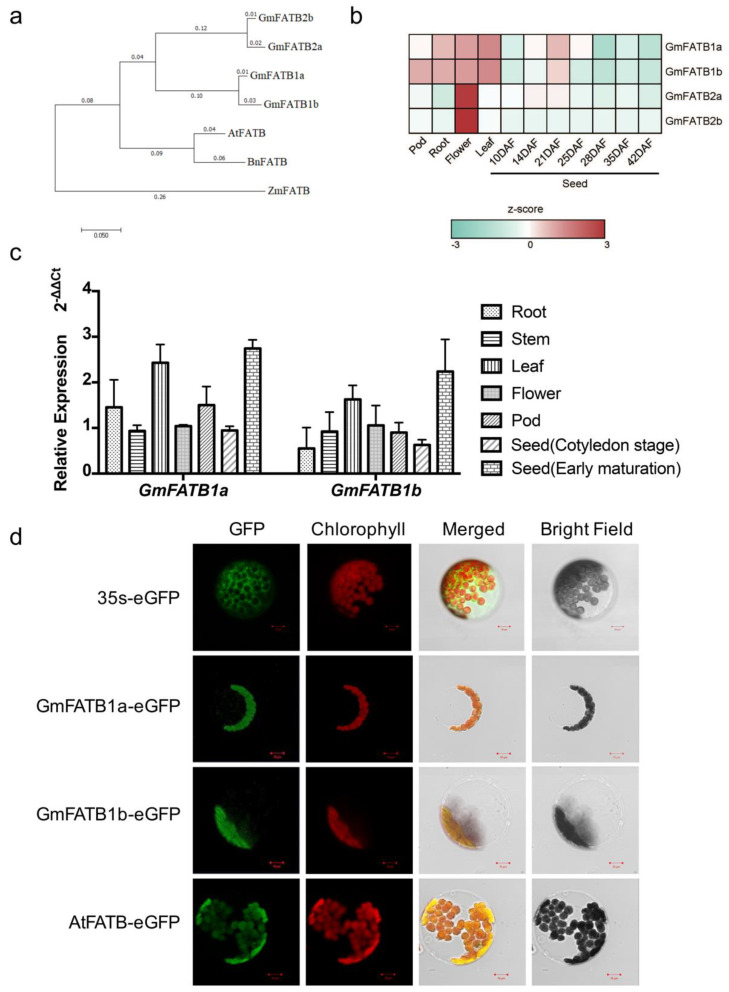
Characterization of the FATBs from soybean. (**a**) Phylogenetic analysis of FATBs from different species. Amino acids sequences were aligned by Clustal W and the phylogenetic tree was constructed based on the construct *Neighbor-Joining* Tree, using MRGA 6.0(At, *Arabidopsis thaliana*; Gm, *Glycine max*; Zm, *Zea mays*; Bn, *Brassica napus*). (**b**) *GmFATBs* expression pattern diagram. Using TBtools software, the z-score further normalized the FPKM value of each gene in different tissue samples and we then drew a heat map. (**c**) *FATB1a* and *FATB1b* were constitutively expressed. Total RNA from soybean Williams 82 was extracted from different tissue. Seeds at the cotyledon stage correspond to 10–14 days after fertilization. The early maturation stage is represented by 15–20 days after fertilization. The transcript levels were detected by qRT-PCR and calculated relative to *GmACTIN* (Glyma.08G146500). The relative expression level was calculated using the transcript abundance of the gene in tissue of “Stem”. The left panel is *GmFATB1a* (Glyma.05G012300). The right panel is *GmFATB1b* (Glyma.17G012400). (**d**) Subcellular localization of GmFATB1a/1b in *Arabidopsis* protoplasts. Green represents the fusion protein fluorescence, and red represents chloroplast autofluorescence. Scale bar = 10 μm.

To better understand the role of the soybean FATB proteins on the soybean oil profile, a BLAST search was performed against the soybean genome database (https://phytozome.jgi.doe.gov/pz/portal.html, accessed on 9 October 2016) [35], using the amino acid sequences of AtFATB. A total of four FATB proteins were found, which can be divided into two pairs, namely GmFATB1a and GmFATB1b, and GmFATB2a and GmFTAB2b. Phylogenetic analysis of the amino acid sequences of FATBs from *Arabidopsis thaliana*, *Brassica napus* (rapeseed), *Zea mays* (maize), and *Glycine max* (soybean) showed that the two pairs of soybean FATBs, i.e., GmFATB1a, GmFATB1b, GmFATB2a and GmFATB2b, are closely related (Figure 1a). Sequence similarity analysis showed that the sequence similarity between the soybean FATB proteins with AtFATB was 82.0%, 82.9%, 78.9%, and 78.4%, respectively (Figure S1b).

The expression patterns of these *GmFATB* genes were examined using a published RNA-seq dataset (https://www.soybase.org/soyseq/, accessed on 8 November 2016) [36] and confirmed by quantitative reverse-transcription PCR (qRT-PCR) (Figure 1b,c). Both the online RNA-seq and qRT-PCR results showed that *GmFATB1a*, *GmFATB1b*, *GmFATB2a*, and *GmFATB2b* were expressed in all soybean tissues tested. *GmFATB1a* and *GmFATB1b* were expressed at higher levels in leaves and seeds (Figure 1b,c), while *GmFATB2a* and *GmFATB2b* were highly expressed in the flowers (Figure S2). *GmFATB1a* and *GmFATB1b* were selected for further study due to their higher expression levels in seeds. The amino acid sequences of GmFATB1a and GmFATB1b displayed 96% sequence similarity (Figure S1).

To determine the subcellular localization of GmFATB1a and GmFATB1b, genomic DNA sequences of *GmFATB1a* and *GmFATB1b* were fused to the N-terminus of the eGFP protein and transiently expressed in *Arabidopsis* mesophyll protoplasts. AtFATB was used as a positive control, while a blank vector was transiently expressed in the *Arabidopsis* protoplasts as a negative control. As shown in Figure 1d, the florescent signals of both the GmFATB1a-eGFP and GmFATB1b-eGFP fusion proteins were observed in the chloroplast, similar to the reported chloroplast localization of AtFATB [37].

### 2.2. Knock-Out of GmFATB1a and GmFATB1b by CRISPR/CAS9

**Figure 2:**
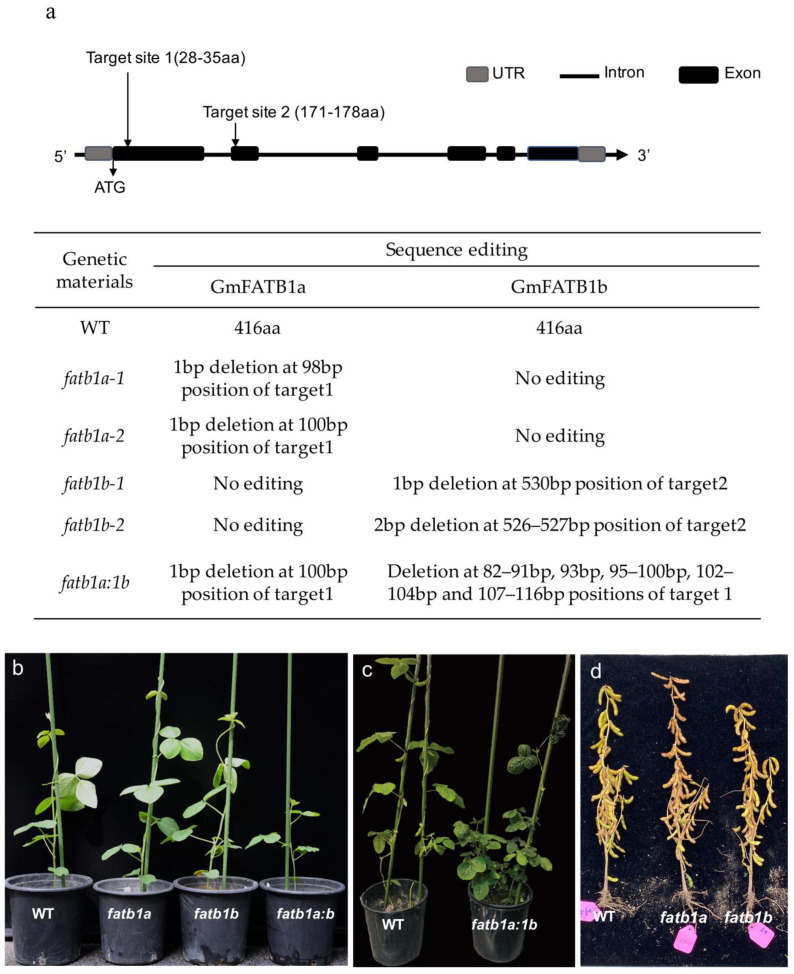
Identification of the mutant lines produced by the CRISPR-Cas9 system. (**a**) Gene structure and sequence targeted for alteration. The mutant sites are listed by form. (**b**) The growth characteristics of the mutant lines. The double mutant *fatb1a:1b* was overall shorter than the wild type, *fatb1a* and *fatb1b*, 21 days after germination, while no difference was observed with the single mutants. (**c**) The double mutant *fatb1a:1b* was overall shorter than the wild type. (**d**) The plant height of the wild type, *fatb1a* and *fatb1b*, were the same in the field experiment. WT: wild type.

FATs are known as FA biosynthesis key enzymes that can release free FAs and acyl-ACP, which determines the length of the carbon chain and ultimately affects the FA composition. To gain an understanding of the function of the GmFATB1 in vivo, we generated mutants that knocked out either or both of the *GmFATB1* genes through CRISPR/Cas9 technology. *GmFATB1a* and *GmFATB1b* have the same gene structure with 6 exons and 5 introns. We selected two sites as CRISPR targets: Target Site 1, which was in the same region of both *GmFATB1a* and *GmFATB1b* and located on the first exon, and Target Site 2, which was located on the second exon (Figure 2a). Single and double mutations of the two genes were obtained, including four single mutant lines of *GmFATB1a* or *GmFATB1b*, named *fatb1a-1* and *fatb1a-2*, and *fatb1b-1* and *fatb1b-2*, respectively. For *fatb1a-1* and *fatb1a-2*, the mutants had an A deletion and a C deletion in the first exon, respectively. In contrast, *fatb1b-1* and *fatb1b-2* had a T deletion and CT deletion in the second exon, respectively. The double mutation of *GmFATB1a* and *GmFATB1b*, *fatb1a:1b*, had a C deletion in *fatb1a* and 30 bp deletion in *fatb1b* in the first exon (Figure 2a).

There was no significant difference observed among the wild type and single mutants at 21 days after germination, when the soybean plants had five true leaves, while the double mutant *fatb1a:1b* showed a significant growth defect compared to the wild type (Figure 2b). During the filling stage, the double mutant *fatb1a:1b* was overall shorter than the wild type and showed male sterility (Figure 2c). At the maturation stage, the plant heights of these single mutants were the same as that of the wild type (Figure 2d). The double mutant *fatb1a:1b* plants grew continuously slower and were male sterility.

### 2.3. Effect of GmFATB1a and GmFATB1b on Leaf FA Profiles

In order to examine the function of the GmFATB1 in plant development, the leaf FA profiles of *fatb1a*, *fatb1b*, and *fatb1:1b* were analyzed. The second trifoliate leaf from the top was sampled for detection of the FA content through gas chromatography. While the content of palmitic acid in the wild type was 6.89 mg/g in the leaves, those of the four single mutant lines of *fatb1a* and *fatb1b* were reduced to 5.43–6.08 mg/g, which ranged from an 11 to 21% reduction. The leaf palmitic acid content of the double mutant *fatb1a:1b* reduced to 3.98 mg/g, which was a 43% reduction compared to the wild type (Table 1 and Table S1). Furthermore, the stearic acid content of the *fatb1a* and *fatb1b* mutants were reduced from the wild type from 2.4 mg/g to 1.89–2.13 mg/g, which is an 11–21% reduction. The stearic acid content of *fatb1a:1b* was 1.55 mg/g, which is a 35% reduction compared to the wild type (Table 1 and Table S1). There were no significant changes in the leaf unsaturated FAs, oleic acid, linoleic acid, and linolenic acid in *fatb* mutants.

### 2.4. Double Mutation of GmFATB1 Causes Male Sterility

**Figure 3:**
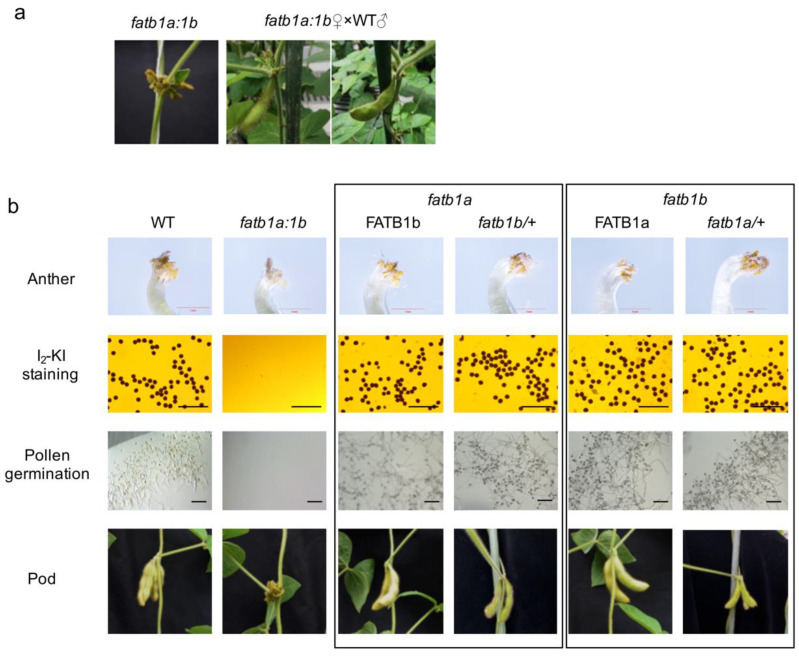
Double mutation of *GmFATB1* leads to male sterility. (**a**) Successful seed sets (right two images) of *fatb1a:1b* after pollination with the WT pollens compared with the unfertilized *fatb1a:1b* pods (left image). (**b**) Processes of pollen and seed development. Anther, scale bar = 1 mm. I_2_-KI staining, scale bar = 200 μm. Pollen germination, scale bar = 200 μm. WT, wild type.

Under both greenhouse and field conditions, the *GmFATB1* double mutation, *fatb1a:1b*, did not set seed (Figure 3a). To evaluate the cause, we first examined whether the development of male/female reproductive systems of the *fatb1a:1b* was abnormal by crossing with the wild type. When pollinated with the wild type pollen, the *fatb1a:1b* plants can set seeds (Figure 3a), indicating that the mutants have a defect only in the male reproductive parts. Dissecting the *fatb1a:1b* flowers showed that the *fatb1a:1b* anther was transparent and lacked pollen (Figure 3b). Scanning electron microscopy (SEM) analysis revealed that the surface of the anther of the *fatb1a:1b* was rough and wrinkled, and no pollen could be found (Figure S3a). Semi-thin sectioning and transmission electron microscopy (TEM) showed that the anther development of the *fatb1a:1b* was abnormal (Figure S3b,c).

Iodine potassium iodide (I_2_-KI) staining of pollens from the heterozygous plants, *fatb1a/+: fatb1b* and *fatb1a: fatb1b/+*, which contained at least one functional allele in *GmFATB1a* and *GmFATB1b*, confirmed that the heterozygous plants can produce viable pollen. Furthermore, in vitro pollen germination showed that the pollen of the heterozygous plants had normal viability and germinability (Figure 3b).

### 2.5. Mutation of GmFATB1 Significantly Improves the Soybean Nutritional Quality

**Figure 4:**
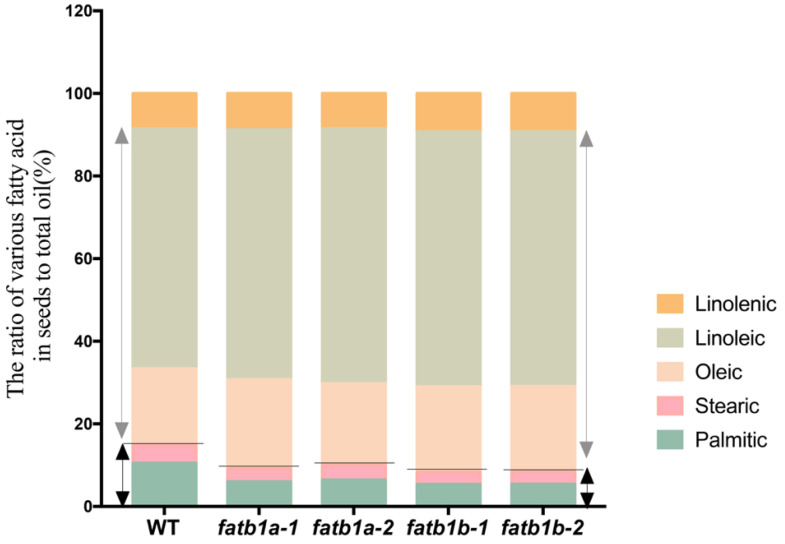
The ratio of the fatty acids in seeds to total oil. The proportions of various fatty acids are shown in the figure. The five colors in the picture represent different types of fatty acids. Below the solid gray lines represent saturated fatty acids, and above the solid gray lines represent unsaturated fatty acids. The black double arrows represent saturated fatty acids, and the gray double arrows represent unsaturated fatty acids. WT, wild type.

To investigate the effect of GmFATB1 on seed FA profile, the FA content in *fatb1a* and *fatb1b* were measured using the T_5_ generation of the CRISPR/CAS mutant seeds produced in large field plots. The result showed that the palmitic acid content of the wild type seeds was 22.85 mg/g, while that in the *fatb1a* and *fatb1b* mutants were reduced to 10.64–13.93 mg/g, which were nearly half that of the wild type (Table 2). Similarly, the stearic acid content of the wild type seed was 9.42 mg/g, while that in the *fatb1a* and *fatb1b* mutants were reduced to 5.92 to 7.79 mg/g (Table 2), which were 63–83% of the wild type (Table S2). No significant change was found in the seeds’ unsaturated FAs between the wild type and the mutants. As the content of the saturated FAs decreased, the total oil content in these mutants decreased, which in turn increased the relative content of the oleic and linoleic acid, which are beneficial to human health (Figure 4). Compared to the wild type, the content of oleic and linoleic acid increased from 18% to 21% and from 58% to 61%, respectively, while the content of the saturated FAs decreased from 14% to 8%. No significant difference was found in the protein content between the mutant and wild type (Table S3).

To determine if there was any yield penalty associated with these changes, wild type and T_5_ generations of the *fatb1a*, and *fatb1b* gene-edited plants were grown in the field to evaluate their agronomic traits, including plant height, number of brunches, number of pods/plant, number of seeds/plant, seed weight/plant, and 100-seed weight in field conditions. The result showed that growth differences compared to the wild type did not exist. The agronomic traits were the same in *fatb1a*, *fatb1b*, and the wild type (Table 3).

## 3. Discussion

CRISPR/Cas-mediated genome editing technology was used to inactivate either of the soybean *FATB1a* or *FATB1b* genes, and the analyses revealed a significant reduction in the content of saturated FAs (Table 1; Table 2) and a relative increase in the linoleic acid content without imposing a yield penalty (Table 3). These results were verified for four generations in both greenhouse and field conditions. The alteration of the FA profiles meets the soybean breeding goal of producing oil that is beneficial for human health (Figure 4). After selection of segregants free of the CAS9 protein and other elements dragged from the transformation vectors, the *fatb1a* and *fatb1b* mutants can be used as breeding germplasm with less safety concerns.

Reports of FATB function in other plants are limited to *Arabidopsis* and maize. In *Arabidopsis*, the FATB function was evaluated in leaves and mainly focused on the synthesis pathway and metabolic reaction, including the changes in eukaryotic and prokaryotic glycerolipid contents and the rate of fatty acid synthesis and degradation [29,30]. In maize, FATB mainly played roles in the reduction of palmitic acid content in the seeds; whether it affects the content of saturated FAs in other tissues is unknown [32]. Unlike the knockout of *Atfatb*, the content of unsaturated FA did not change in leaves and seeds of the soybean *fatb1a* and *fatb1b* mutants. This may be due to the dynamic balance of the lipid synthesis regulatory network. Soybean seeds are rich in oil, which occupies an important position in the world’s vegetable oil consumption and inactivation of *FATB1a* or *FATB1b* resulted in a reduced content of saturated soybean oil, which is desirable for nutritional purposes. Thus, the function of FATB in soybean is more important for agronomic purposes than in other species studied to date. A soybean mutant line with a 254kb-deletion, including 19 functional genes, showed a low seed palmitic acid content phenotype [34], consistent with the findings of this study. Although it was speculated that FATB1a may play an important role in reducing the palmitic acid content of seeds, there was no direct evidence or description of other functions of FATB1 and the role of its homologous gene *FATB1b* was also unclear [34]. Here, we identified two *FATB* homologous genes in soybean, and for the first time directly showed that they carry out similar functions in determining the content of palmitic acid and stearic acid in soybean.

It was reported that seed-specific expression of *AtFATB* can significantly increase the content of palmitic acid in *Arabidopsis* seeds [31]. In our study, *GmFATB1* overexpression plants only slightly increased the palmitic content and had no effect on the stearic acid content (Figure S4, Table S4). This difference may be caused by the additional acyltransferase(s) in soybean, counteracting the effect of the elevation of the *GmFATB1* expression. It is also possible that a counterbalance mechanism to avoid overaccumulation of saturated FAs is triggered by increasing the degradation rate of the saturated FAs in soybean.

In *Arabidopsis*, mutants of many different genes have been identified to result in abnormal pollen development and male sterility due to the reduced FA content [38,39,40,41]. However, male sterility as a result of the alteration of the FA content has not been reported in soybean. Double mutant *fatb1a:1b* was completely male sterility, which is the first evidence to relate male sterility with an imbalance in the soybean FA profile. Palmitic acid and steric acid are precursors for the synthesis of cutin, wax, sporopollenin, and tryphine [42,43]. FAs and their derivatives are essential building blocks for anther cuticle and pollen-wall formation. Disruption of lipid metabolism during anther and pollen development often leads to genic male sterility, in *Arabidopsis* and maize [44]. The large reduction in saturated FAs could not meet the demand for FA content in soybean anthers during flowering, leading to failure of pollen development and male sterility of *fatb1a:1b*.

Our work highlights the effect of GmFATB1a and GmFATB1b in determining the oil profile in soybean and provides a foundation for improving soybean oil quality by directed molecular breeding. Research has shown that soybean domestication generated thriving genetic variations in soybean germplasm [45,46,47]. Identification of the superior alleles of *GmFATB1a* and *GmFATB1b* would also be valuable resources for the genetic improvement of seed oil quality in soybean-breeding programs. In addition, a genetic approach to stack *fatb1a* and *fatb1b* with other genes involved in FA synthesis may further improve the soybean oil profile [48,49]. The use of emerging editing systems that can edit multiple genes simultaneously with removal of selection markers would provide material that can be integrated into breeding programs.

## 4. Materials and Methods

### 4.1. Plant Materials and Growth Conditions

Soybean (*Glycine max*) cultivar (Williams 82) was used as the wild type control, and transformation was performed on this cultivar. Soybean seeds were germinated and transplanted in pots filled with the matrix media (Pindstrup Mosebrug A/S, Shanghai, China). Soybean plants were grown in a greenhouse at 30/24 °C with a photoperiod of 16/8 h and a relative humidity of about 70–80%. For the field trials, soybean plants were grown at the Anhui Academy of Agricultural Science (Hefei, China) (E 117.25, N 31.89).

### 4.2. Vector Construction and Soybean Transformation

The CRISPR/Cas9 construct was based on the pBlu-gRNA vector and Cas9 MDC123. The targets were designed using the online tool CRISPR v2.0 (http://crispr.hzau.edu.cn/cgi-bin/CRISPR2/CRISPR, accessed on 14 November 2016) based on their GC content and off-target efficiency. The 20-bp target sequence (5′-GTTAAAAGTGCTGGGCTTCTTGG-3′) in the first exon of the coding sequence was designed as Target 1, while the other 20-bp target sequence (5′-GTTAAAAGTGCTGGGCTTCT -3′) in the second exon was designed as Target 2. These target sequences were synthesized and cloned into the pBlu-gRNA vector at the *Bbs*I site under the control of the U6 promoter. The constructs were then digested with *Eco*RI to produce the gRNA cassette, which was then inserted into Cas9 MDC123. The final constructs were named *fatb1*-Cas9-1 and *fatb1*-Cas9-2.

In addition, we used the Gm*FATB1a* endogenous promoter to overexpress Gm*FATB1*. A fragment containing a 1725-bp promoter of *GmFATB1a* (ATG upstream of the gene), a TATA region, an Omega sequence, a Flag tag, and a 2999-bp coding region sequence of *GmFATB1a* (Glyma.05G012300) was cloned into the PTF101 vector. The binary vector was named GmFATB1-OE.

The above binary vectors were introduced into an *Agrobacterium* strain LBA4404. Transgenic soybeans were obtained via *Agrobacterium*-mediated soybean transformation using the cotyledonary node explant and the herbicide resistance gene *bar* as a selection marker, as described in [50]. Soybean variety Williams 82 was used as the transformation recipient. The homozygous mutants of *GmFATB1a* or *1b*, and the double gene mutant, were screened at T_0_ generations after sequencing of the target sites.

To observe subcellular localization of FATB1a and FATB1b, the PC1300 vector driven by the cauliflower mosaic virus (CaMV) 35S promoter was used. The genomic DNA of *FATB1a* (2525 bp) and *FATB1b* (2515 bp) without stop codons were cloned into the construct by GBclonart (Genebank Bioscience Inc, Suzhou, China) and fused to the N-terminus of eGFP. The final binary vectors, *P_35S_:GmFATB1a-eGFP* and *P_35S_:GmFATB1b-eGFP*, were used to observe the subcellular localization of GmFATB1a and GmFATB1b.

### 4.3. Phylogenetic Tree Construction

A phylogenetic tree was constructed by the *Neighbor-Joining* method of Mega version 6.0 [51], by using the amino acid sequence of the FATB protein of *Glycine max* (soybean), *Arabidopsis thaliana* (*Arabidopsis*), *Zea mays* (maize), and *Brassica napus* (rapeseed). The similarity and identity analysis were done on the website https://www.ebi.ac.uk/Tools/psa/emboss_needle/ (accessed on 8 November 2016).

### 4.4. RNA Extraction and qRT-PCR

Total RNA from different tissues, which were collected from soybean plants potted in the soil in the greenhouse, was isolated following the instructions of a plant RNA Extract Kit (TIANGEN Biotech, Beijing, China). The cDNA was synthesized with the Prime Script RT Master Mis kit (Takara, Shiga, Japan). The qRT-PCR was conducted using SYBR premix Ex Taq (RR420, Takara, Japan) with a LightCycler^®^ 480 machine (Roche, Germany). *GmACTIN* was used as internal control. The relative expression level was calculated using the formula 2^−Δ(Δct)^ [52]. For the quantification of each gene, at least three biological replicates and two technology replicates were used. Primers for qRT-PCR analysis were given in Table S5.

### 4.5. Subcellular Localization in Arabidopsis Mesophyll Protoplasts

To observe the subcellular localization of GmFATB1a, GmFATB1b, and AtFATB, the plasmids fused with the target genes and GFP were transfected into *Arabidopsis* mesophyll protoplasts. The method was performed as previously described [53]. After harvesting protoplasts, autofluorescence signals of GFP and chlorophyll were observed under an LSM 710 NLO confocal laser scanning microscope (Zeiss, Jena, Germany).

### 4.6. Pollen Grain Staining

In order to study the development of pollen, pollen grains were immersed in 1% (*v*/*v*) I_2_-KI solution for 20 min at room temperature and pollen grains observed using a microscope. Normally developed pollen rich in starch was stained black with I_2_-KI solution, while dysplastic pollen was often deformed, and usually did not contain starch or had a low starch content, and was usually yellow-brown. Pollen fertility was judged based on these criteria.

### 4.7. Scanning Electron Microscopy (SEM)

Soybean flowers that were about to bloom were used as samples for the scanning electron microscopy and were treated as follows: (1) Double fixation: The sample was first fixed with 2.5% (*v*/*v*) glutaraldehyde in phosphate buffer (0.1 M, pH7.0) for more than 4 h and washed three times in a phosphate buffer (0.1 M, pH7.0) for 15 min at each step; then, secondly, fixed with 1% (*v*/*v*) OsO4 in a phosphate buffer for 1–2 h and washed three times in the phosphate buffer (0.1 M, pH7.0) for 15 min at each step. (2) Dehydration: The sample was first dehydrated by a graded series of ethanol (30% (*v*/*v*), 50%, 70%, 80%, 90%, and 95%) for about 15 min at each step, then dehydrated twice by the above alcohol concentration for 20 min at each step or stored in alcohol. (3) The sample was dehydrated in Hitachi Model HCP-2 critical point dryer. (4) Coating and observation: The dehydrated sample was coated with gold–palladium in a ion sputter (Hitachi Model E-1010, HITACHI, Toyoto, Japan) for 4–5 min and observed in a SEM (Hitachi Model SU-8010, Japan).

### 4.8. Transmission Electron Microscopy (TEM)

Flowers at a similar development stage as used for the scanning electron microscopy were selected and treated: (1) Double fixation: The sample was first fixed with 2.5% glutaraldehyde in phosphate buffer (0.1 M, pH7.0) for more than 4 h and washed three times in a phosphate buffer (0.1 M, pH7.0) for 15 min at each step; then, secondly, fixed with 1% OsO_4_ in a phosphate buffer for 1–2 h and washed three times in the phosphate buffer (0.1 M, pH7.0) for 15 min at each step. (2) Dehydration: The sample was first dehydrated by a graded series of ethanol (30%, 50%, 70%, and 80%) for about 15 min at each step, and then dehydrated by a graded series of acetone (90% and 95%) for about 15 min at each step; and, finally, dehydrated twice by absolute acetone for 20 min. (3) Infiltration: The specimen was placed in a 1:1 mixture of absolute acetone and the final Spurr resin mixture for 1 h at room temperature, then transferred to a 1:3 mixture of absolute acetone and the final resin mixture for 3 h, and finally to the Spurr resin mixture overnight. (4) Embedding, ultrathin sectioning, staining, and observation: A specimen was placed in an Eppendorf tube contained Spurr resin and heated at 7 °C for more than 9 h. The specimen was sectioned using a LEICA EM UC7 ultratome and sections were stained by uranyl acetate and alkaline lead citrate for 5 to 10 min, respectively, and observed in a TEM (Hitachi H-7650, Japan).

### 4.9. FA Analysis

For the determination of the FA analysis, as mentioned above [54], quantitative analysis was performed by heating the methylation extraction method and gas chromatography analysis (Agilent, CA, USA, a DB-23 column). The tissue samples were dried and grounded into powder for subsequent fatty acid extraction. First, we weighed 50 mg of the sample into a 15 mL glass tube, and then added 2 mL of extraction solution (chloroform: isopropanol (*v*/*v*) = 2:1) and extracted for 2 h in the dark. Next, we added 2 mL of a 1% sulfuric acid methanol solution (*v*/*v*) and heated it at 80 °C for 1 h to carry out the methyl esterification reaction. Finally, fatty acid methyl ester (FAME) was extracted with 3 mL hexane and 1 mL 0.9% (*w*/*v*) NaCl. The 1 mL extract was passed through a gas chromatograph for FAME detection. The detection procedure includes three procedures: (1) 120 °C for 5 min; (2) raise the temperature to 190 °C and protect for 12 min, the heating rate is 4 °C per min; and (3) raise the temperature to 210 °C and keep at the temperature for 10 min, the heating rate is 2.5 °C per min. Finally, we detected at 280 °C using a gas chromatography–flame ionization detector (7890A, Agilent, USA).

### 4.10. Seed Protein Analysis

The seed protein content was determined by the modified Kjeldahl method. The detailed steps are described in the literature [54]. Samples with 200 mg heat-dried seed powder were digested by 15 mL H_2_SO_4_ and a composite catalyst (CuSO4 and K_2_SO4) overnight. Then, the samples were treated as follows: 160 °C, 15 min; 220 °C, 30 min; 350 °C, 30 min; and 450 °C, 120 min. The nitrogen content was analyzed using an automatic Kjeldahl apparatus (KjeltecTM 8400, FOSS, Denmark). The relative protein content (%) was calculated by multiplying the percentage of nitrogen content by a factor of 6.25.

## Figures and Tables

**Table 1:** Leaf fatty acid content of the different materials.

Material (mg/g)	Palmitic	Stearic	Oleic	Linoleic	Linolenic	Total
Wild type	6.89 ± 0.21 a	2.40 ± 0.06 a	0.73 ± 0.01 a	4.22 ± 0.10 a	33.52 ± 2.36 a	47.76 ± 2.03 a
*fatb1a-1*	6.08 ± 0.42 b	2.13 ± 0.05 b	0.76 ± 0.11 a	4.29 ± 0.19 a	34.01 ± 0.32 a	47.27 ± 0.21 a
*fatb1a-2*	5.66 ± 0.34 bc	1.98 ± 0.06 bc	0.73 ± 0.12 a	3.91 ± 0.54 a	31.64 ± 3.04 a	43.93 ± 2.46 b
*fatb1b-1*	5.86 ± 0.14 bc	2.05 ± 0.09 bc	0.87 ± 0.19 a	4.34 ± 0.45 a	32.53 ± 0.66 a	45.65 ± 1.42 ab
*fatb1b-2*	5.43 ± 0.12 c	1.89 ± 0.05 c	0.73 ± 0.04 a	4.29 ± 0.22 a	31.26 ± 1.08 a	43.59 ± 1.44 b
*fatb1a: 1b*	3.98 ± 0.22 d	1.55 ± 0.11 d	0.86 ± 0.16 a	4.56 ± 1.00 a	35.21 ± 2.38 a	43.88 ± 2.09 b

Data are given as the mean ± SD (*n* = 3). Different letters indicate significant differences (least significant difference test, *p* < 0.05).

**Table 2:** Seed fatty acid content of the different materials (mg/g).

Materials	Saturated Fatty Acid (mg/g)			Unsaturated Fatty Acid (mg/g)				Total (mg/g)
	Palmitic	Stearic	Total	Oleic	Linoleic	Linolenic	Total	
Wild type	22.85 ± 0.98 a	9.42 ± 0.56a	32.28 ± 1.53a	40.45 ± 1.70 bc	126.73 ± 6.09 ab	18.72 ± 0.93 ab	185.89 ± 8.56 a	218.17 ± 10.09 a
*fatb1a-1*	12.23 ± 0.82 b	6.43 ± 0.44 c	18.66 ± 1.26 c	43.97 ± 4.08 a	123.51 ± 4.53 b	17.90 ± 1.44 c	185.38 ± 6.53 a	204.04 ± 6.13 b
*fatb1a-2*	13.93 ± 0.30 c	7.79 ± 0.18 b	21.72 ± 0.43 b	42.72 ± 1.63 ab	133.66 ± 4.50 a	18.47 ± 0.52ab	194.85 ± 6.62 a	216.57 ± 7.05 a
*fatb1b-1*	10.81 ± 1.17 d	6.06 ± 0.50 c	16.87 ± 1.61 cd	41.75 ± 4.76 bc	125.00 ± 10.80 ab	18.81 ± 1.07 ab	185.55 ± 16.41 a	202.42 ± 17.14 b
*fatb1b-2*	10.64 ± 0.84 d	5.92 ± 0.16 c	16.56 ± 0.99 d	40.54 ± 3.62 bc	121.27 ± 7.38 b	18.23 ± 0.48 b	180.04 ± 11.26 a	196.60 ± 10.73 b

Total: total fatty acid content. Data are given as the mean ± SD (*n* = 3). Different letters indicate significant differences (least significant difference test, *p* < 0.05).

**Table 3:** Agronomic performance of the wild type, *fatb1a*, and *fatb1b* homozygous genetically modified material plants.

Material	Plant Height (cm)	Number of Branches	Number of Pods/Plant	Number of Seeds/Plant	Seed Weight (g)/Plant	100-Seed Weight (g)
Wild type	53.3 ± 3.6 a	2.7 ± 0.5 a	57.6 ± 8.5 a	127.1 ± 17.9 a	12.8 ± 2.9 a	13.3 ± 1.6 a
*fatb1a-1*	53.5 ± 4.7 a	3.5 ± 1.5 a	64.9 ± 23.4 a	130.7 ± 44.7 a	12.9 ± 4.6 a	13.7 ± 1.0 a
*fatb1a-2*	50.2 ± 4.7a	2.8 ± 0.8 a	63.1 ± 17.4 a	140.5 ± 41.3 a	14.6 ± 3.0 a	13.0 ± 1.4 a
*fatb1b-1*	47.3 ± 9.8 a	2.6 ± 0.8 a	56.3 ± 16.1 a	114.1 ± 43.4 a	13.2 ± 1.3 a	13.2 ± 1.3 a
*fatb1b-2*	50.0 ± 6.1 a	2.6 ± 1.2 a	69.4 ± 19.7 a	107.5 ± 31.6 a	13.9 ± 4.8 a	12.7 ± 1.0 a

Data are given as the mean ± SD (*n* = 10). Different letters indicate significant differences (least significant difference test, *p* < 0.05).

## Data Availability

Data is contained within the article or Supplementary Materials.

## Supplementary Materials

Supplementary Materials can be found at https://www.mdpi.com/article/10.3390/ijms22083877/s1. Figure S1. Alignment of FATBs protein sequence. Figure S2. Expression pattern of *GmFATB2a* and *GmFATB2b*. Figure S3. Pollen morphology. Figure S4. Expression of *GmFATB1a* relative to *GmACTIN* in the overexpression lines. Table S1. The ratio of leaf fatty acid content of various mutants to the wild type (%). Table S2. The ratio of seed fatty acid content of various mutants to the wild type (%). Table S3. The ratio of various fatty acid in seeds to total oil (%) and protein content (%). Table S4. Fatty acid content in leaf and seed of OE materials. Table S5. Primers used in this study.

## Abbreviations

ACP	acyl carrier protein
FA	fatty acid
FATA(B)	acyl-acyl carrier protein thioesterase A(B)
I_2_-KI	iodine potassium iodide
LDL-C	low-density lipoprotein cholesterol
SEM	scanning electron microscopy
TEM	transmission electron microscopy
