# Supplementary material for: CRISPR/Cas9-Mediated Knockout of GmFATB1 Significantly Reduced the Amount of Saturated Fatty Acids in Soybean Seeds
Source: Int J Mol Sci. 2021 Apr 9;22(8):3877. doi: 10.3390/ijms22083877 (PMC8069101; doi:10.3390/ijms22083877)
Supplement: Supplementary file 1 [file ijms-22-03877-s001.pdf]

Supplementary Materials

a

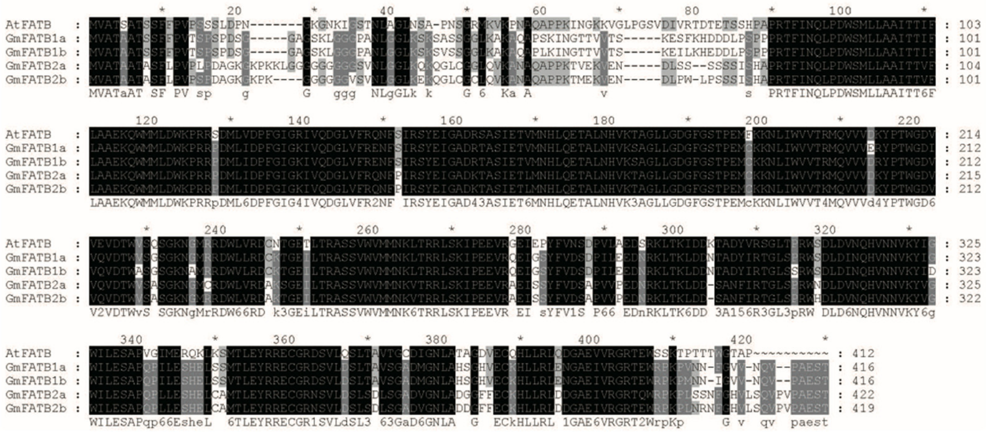

b

	AtFATB	GmFATB1a	GmFATB1b	GmFATB2a	GmFATB2b
AtFATB	—	73.8%	73.9%	69.7%	68.1%
GmFATB1a	82.0%	—	96.4%	76.7%	77.5%
GmFATB1b	82.9%	97.8%	—	75.1%	76.8%
GmFATB2a	78.9%	85.1%	83.5%	—	95.3%
GmFATB2b	78.4%	85.4%	84.3%	96.7%	—

Figure S1. Alignment of FATBs protein sequence. (**a**) Alignment of the amino acid sequences of five FATBs from *Arabidopsis* (AtFATB) and soybean (GmFATB). The black and grey shading indicates identical and similar sequences, respectively. (**b**) Identity and similarity matrix for different species FATB proteins. The blue and orange background represent similarity and identity, respectively.

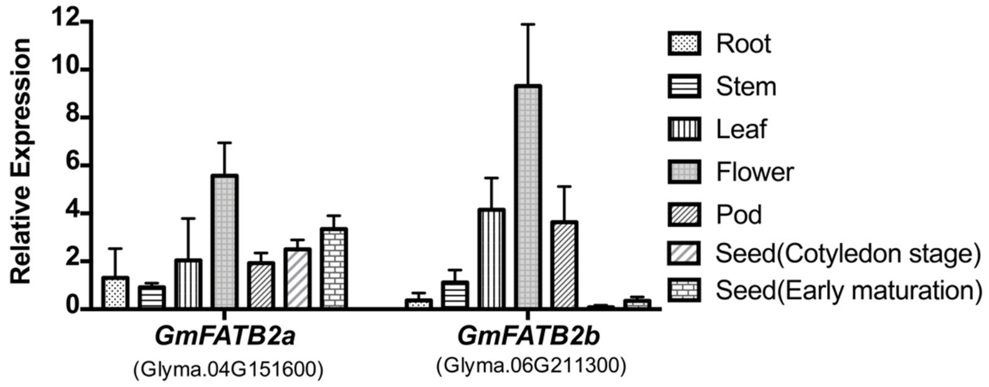

Figure S2. Expression pattern of *GmFATB2a* and *GmFATB2b*. Total RNA from soybean Williams 82 was extracted from different tissue. Seeds at the cotyledon stage correspond to 10-14 days after fertilization. Early maturation stage represents 15-20 days after fertilization. Transcript levels were determined by qRT- PCR and calculated relative to *GmACTIN* (Glyma.08G146500). The relative expression level was calculated using the formula $2^{-\Delta(\Delta ct)}$. The left panel shows *GmFATB2a* (Glyma.04G151600). The right panel shows *GmFATB2b* (Glyma.06G211300).

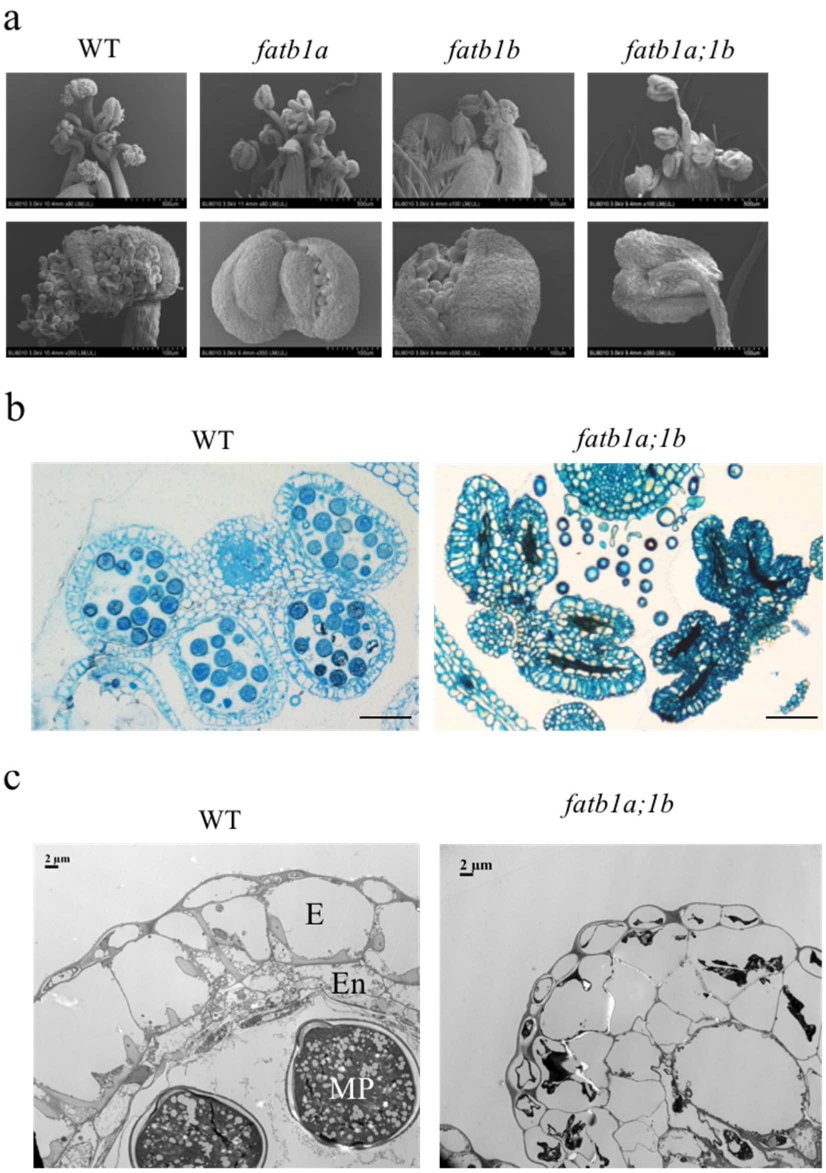

Figure S3. Pollen morphology. (**a**) Scanning electron microscope (SEM) of pollen from wild type, *fatb1a*, *fatb1b* and *fatb1:1b*. (**b**) Semi-thin section comparison of mature anther in the wild type and *fatb1a:1b*. Transverse section of wild type and *fatb1a:1b* anthers were stained with toluidine blue. The image is a cross-section through a single chamber, and the picture is captured using a 20X optical microscope. Scale bar = 50 µm. (**c**)Transmission electron microscope (TEM) of pollen from wild type and *fatb1a:1b*. These images showed the structure of anther wall. E, epidermis; En, endothecium; MP, mature pollen. Scale bar = 2µm.

WT: wild type

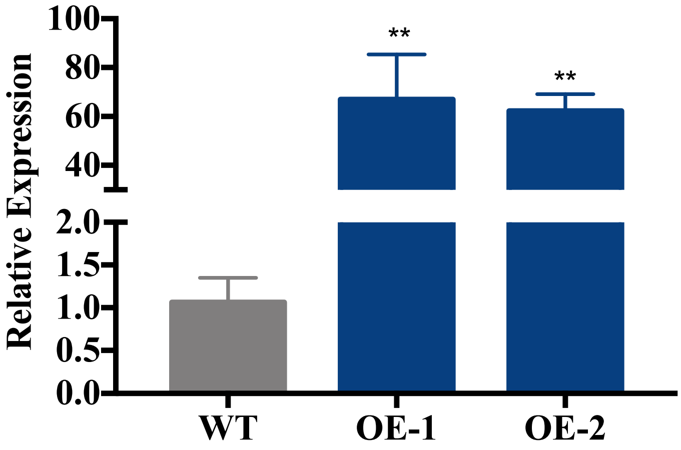

Figure S4. Expression of *GmFATB1a* relative to *GmACTIN* in the overexpression lines. Gm*FATB1a* was overexpressed by introducing an additional copy of the *GmFATB1a* genomic sequence driven by its native promoter and omega enhancer. Two independent transgenic lines were generated and designated OE-1, and OE-2. WT: wild type. Data are means (n = 3). * $p < 0.05$, ** $p < 0.01$. (One-way ANOVA followed by Dunnett's multiple comparisons test).

Table S1. The ratio of leaf fatty acid content of various mutants to wild-type (%).

Material	Palmitic (%)	Stearic (%)	Oleic (%)	Linoleic (%)	Linolenic (%)	Total (%)
Wild type	100.00[1]	100.00[1]	100.00[1]	100.00[1]	100.00[1]	100.00[1]
fatb1a-1	88.25	88.93	103.72	101.63	101.46	98.98
fatb1a-2	82.08	82.67	99.94	92.82	94.40	91.98
fatb1b-1	85.05	85.44	118.59	102.80	97.04	95.57
fatb1b-2	78.73	78.83	99.25	101.83	93.24	91.28
fatb1a; 1b	57.69	64.71	117.15	108.13	98.24	91.86

[1] Consider the content of each fatty acid in the wild-type as 100%. Data are given as means ± SD ($n = 3$).

Different letters indicate significant differences (least significant difference test, $p < 0.05$).

Table S2. The ratio of seed fatty acid content of various mutants to wild-type (%).

Material	Palmitic (%)	Stearic (%)	Oleic (%)	Linoleic (%)	Linolenic (%)	Total (%)
Wild type	100.00[1]	100.00[1]	100.00[1]	100.00[1]	100.00[1]	100.00[1]
fatb1a-1	53.51	68.23	108.70	97.47	95.62	93.52
fatb1a-2	60.94	82.72	105.60	105.47	98.70	99.27
fatb1b-1	47.32	64.29	103.20	98.63	100.50	92.78
fatb1b-2	46.58	62.79	100.22	95.69	97.41	90.11

[1] Consider the content of each fatty acid in the wild-type as 100%. Data are given as means ± SD ($n = 3$). Different letters indicate significant differences (least significant difference test, $p < 0.05$).

Table S3. The ratio of various fatty acid and protein content in seeds (%).

Materials	Saturated fatty acid (%)			Unsaturated fatty acid (%)				Protein content (%)
	Palmitic	Stearic	Total	Oleic	Linoleic	Linolenic	Total	
Wild type	10.47 a	4.32 a	14.79 a	18.54 a	58.09 a	8.58 a	85.21 a	40.46 a
fatb1a-1	5.99 b	3.15 bc	9.14 b	21.55 c	60.53 b	8.77 a	90.86 b	39.95 a
fatb1a-2	6.43 b	3.60 b	10.03 b	19.72 ab	61.72 c	8.53 a	89.97 b	40.58 a
fatb1b-1	5.34 b	2.99 c	8.33 b	20.62 c	61.75 c	9.29 a	91.67 b	40.39 a
fatb1b-2	5.41 b	3.01 c	8.42 b	20.62 c	61.68 c	9.27 a	91.58 b	40.57 a

Data are given as means ± SD (n = 3). Different letters indicate significant differences (least significant difference test, $p < 0.05$).

Table S4 Fatty acid content in leaf and seed of OE materials.

Material tissue (mg/g)	Palmitic	Stearic	Oleic	Linoleic	Linolenic	Total
Leaf						
Wild type	6.89 a	2.40 a	0.73 a	4.22 a	33.52 a	47.76 a
OE-1	6.95 a	2.29 ab	0.69 a	3.85 a	30.45 bc	44.23 b
OE-2	7.06 a	2.36 a	0.67 a	4.17 a	32.66 ab	46.92 a
Seed						
Wild type	22.85 a	9.42 a	40.45 ab	126.73 a	18.72 ab	218.17 a
OE-1	23.69 a	8.89 a	40.09 ab	126.80 a	18.96 ab	218.41 a
OE-2	23.86 a	9.19 a	37.96 b	128.30 a	19.73 a	219.04 a

Data are given as means ± SD (n = 3). Different letters indicate significant differences (least significant difference test, $p < 0.05$).

Table S5. Primers used in this study.

Name	Primer Sequence (5' to 3')
**For qRT-PCR**	
GmFATB1a-qPCR1-F	ATGGGTGGTTACTCGGATGC
GmFATB1a-qPCR1-R	CACGGCGCATACCATTCTTC
GmFATB1b-qPCR-F1	CAGGTTGTGGTGGATCGCTA
GmFATB1b-qPCR-R1	CGTAAAACCCAATCACGGCG
GmFATB2a-qPCR1-F	CTTCAGGAAACTGCTTTG
GmFATB2a-qPCR1-R	TGCTGCATCAACCCAAGT
GmFATB2b-qPCR1-F	ATCATGTCACCAGCTCTG
GmFATB2b-qPCR1-R	TCTTTCCTGCTGCATCGA
**For CRISPR/Cas9 vector construction**	
GmFATB1-Oligo-F1	GATTGGTGGTGGGCCTGCAAACCT
GmFATB1-Oligo-R1	AAACAGGTTTGCAGGCCCACCACC
GmFATB1-Oligo-F2	GATTGTTAAAAGTGCTGGGCTTCT
GmFATB1-Oligo-R2	AAACAGAAGCCCAGCACTTTTAAC
**For overexpression vector construction**	
GmFATB1a-Pro-F	CCACCATGTTGACCTGCACAAATATTTAAATCTTACTGGGG
GmFATB1a-Pro-R	GAGGAAGGGTCTTGCGAAAATGAATTTCTAAGGTCGCTG
GmFATB1a-gDNA-F	GACGACGATGACAAGCATATGGTGGCAACAGCTGCTA
GmFATB1a-gDNA-R	GGATCCTCTAGAGTCGACGCTGGCATAAAATACAAGC
Omega-F	TTCGCAAGACCCTTCCTCTA
Omega-R	ATGCTTGTCATCGTCGTCCT
**For identification of mutations**	
Cas9-identify-F1	TGTTCCTGGCTGCTAAGA
Cas9-identify-R1	ATCTTCTCTCTGTTATCC
GmFATB1a-Cas9-id-F1	ACGTGATCAAATAAGCCTGC
GmFATB1a-Cas9-id-R1	GCTCCAATCAGGCAACTGGT
GmFATB1b-Cas9-id-F1	TACAGTGCTTCTTTGCCT
GmFATB1b-Cas9-id-R1	TCCAATCAGGTAACTGGT
GmFATB1b-Cas9-id-F2	AAATCTGTGTCTTCTGGTGG
GmFATB1b-Cas9-id-R2	TAGCAGCTTAACAAGGGCAC
GmFATB-OE-id-F1	CAGAGGCATCTTCAACGA
GmFATB-OE-id-R1	CTAGTAATGCCAGTACCC

**For subcellular localization**	
AtFATB-GFP-F1	GGACGAGCTCGGTACCCGGGATGGTGGCCACCTCTGCT
AtFATB-GFP-R1	CTCACCATGTCGACTCTAGTCGGTGCAGTTCCCCAAGT
GmFATB1a-EGFP-F1	GGACGAGCTCGGTACCCGGGATGGTGGCAACAGCTGCT
GmFATB1a-EGFP-R1	CTCACCATGTCGACTCTAGGGGTGCTTTCTGCTGGAAC
GmFATB1b-EGFP-F1	GGACGAGCTCGGTACCCGGGATGGTGGCAACAGCTGCA
GmFATB1b-EGFP-R1	CTCACCATGTCGACTCTAGTTAGGTGCTTTCTGCTGG
